# In Situ Detection of Kidney Organoid Generation From Stem Cells Using a Simple Electrochemical Method

**DOI:** 10.1002/advs.202200074

**Published:** 2022-05-04

**Authors:** Intan Rosalina Suhito, Jin Won Kim, Kyeong‐Mo Koo, Sun Ah Nam, Yong Kyun Kim, Tae‐Hyung Kim

**Affiliations:** ^1^ School of Integrative Engineering Chung‐Ang University Seoul 06974 Republic of Korea; ^2^ Cell Death Disease Research Center College of Medicine The Catholic University of Korea Seoul Korea; ^3^ Department of Internal Medicine College of Medicine The Catholic University of Korea St. Vincent's Hospital Suwon Republic of Korea

**Keywords:** electrochemical detection, gold nanostructure, kidney organoid, organoid maturation, pluripotent stem cells

## Abstract

Organoids that mimic the structural and cellular characteristics of kidneys in vitro have recently emerged as a promising source for biomedical research. However, uncontrollable cellular heterogeneity after differentiation often results in the generation of off‐target cells, one of the most challenging issues in organoid research. This study proposes a new method that enables the real‐time assessment of kidney organoids derived from stem cells. When placed on a conductive surface, these organoids generate unique electrochemical signals at ≈0.3 V with intensities proportional to the amount of kidney‐specific cell types. Off‐target cells (i.e., non‐kidney cells) produce an electrical signature at 0 V that is distinguishable from other surrounding cell types, enabling non‐destructive assessment of both the differentiation, and maturation levels of kidney organoids. The developed platform can be applied to other types of organoids and is thus highly promising as a tool for organoid‐based drug screening, toxicity assessment, and therapeutics.

## Graphical abstract

The developed conductive cell culture platform enables the detection of initial pluripotent stem cell numbers and kidney organoid generation in vitro. Both kidney‐specific and off‐target cells are detectable electrochemically in a label‐free and non‐destructive manner. This proposed platform can be extended as a tool for organoid‐based rapid drug screening and is thus highly promising in pharmacological research.

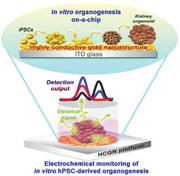

## 1 Introduction

Kidney disease is one of the most complex illnesses and is difficult to treat with current therapeutic methods, resulting in high morbidity, and mortality rates worldwide.^[^ ^1^, ^2^, ^3^ ^]^ In the United States, 15% of adults are diagnosed with chronic kidney disease (CKD). More importantly, the rate of diagnosis has continued to increase over the last two decades.^[^ ^4^ ^]^ Several studies have sought to mimic the complex structure and functions of the kidney in vitro, including the modeling of acquired or genetic kidney diseases, to identify more effective medical treatment techniques and drugs.^[^ ^5^, ^6^, ^7^, ^8^, ^9^, ^10^, ^11^, ^12^ ^]^ Organoids are self‐organized multicellular tissues derived from human pluripotent stem cells (PSCs) in vitro, which have recently emerged as promising models of human disease.^[^ ^13^, ^14^, ^15^, ^16^, ^17^, ^18^, ^19^, ^20^, ^21^ ^]^

In 2015, several researchers reported the successful differentiation of PSCs into nephron‐like structures mimicking the compartments of the kidney.^[^ ^22^, ^23^ ^]^ Kidney organoids generated from PSCs consist of all major renal cell types, including glomerulus, stroma, and endothelial cells, with a distinct tubular structure, which is a critical morphological signature of the kidney.^[^ ^24^, ^25^, ^26^, ^27^, ^28^, ^29^ ^]^ Recent studies have reported that differentiated kidney organoids could potentially be used to develop therapeutic strategies for CKD patients and can also be used as an efficient in vitro model for drug screening and nephrotoxicity testing.^[^ ^30^, ^31^, ^32^, ^33^ ^]^ Although promising, one of the significant drawbacks of kidney organoids is the existence of off‐target cells during and after the differentiation.^[^ ^34^, ^35^, ^36^, ^37^, ^38^ ^]^

The off‐target cells include non‐kidney cell types and undifferentiated PSCs that ultimately result in several critical issues, including the generation of immature organoids, the deformation of their tubular structure, and off‐target cell outgrowth. A recent single‐cell transcriptomic analysis reported that kidney organoids were up to 10%–20% immature due to the presence of nonrenal cells (i.e., off‐target cells).^[^ ^39^ ^]^ Furthermore, organoids cultured under the same conditions (e.g., protocol and experimental setup) often show extreme variations in maturation level.^[^ ^40^ ^]^ Therefore, the rapid and accurate detection of off‐target cells is essential to develop the protocols and methods for the generation of mature kidney organoids with low variations, which is essential for their application both in research and clinical purposes.

Most current techniques for the identification of differentiated cell types (e.g., polymerase chain reaction [PCR], immunohistochemical staining, flow cytometry, and single‐cell RNA sequencing) can effectively confirm the presence of off‐target cells.^[^ ^39^, ^41^, ^42^, ^43^ ^]^ However, unlike the typical two‐dimensional (2D) cell culture, the complex cellular compositions, and microstructures in the three‐dimensional (3D) environment of organoids should be retained during and after the analyses. Therefore, conventional methods that require cell detachment, fixation, dissociation, and lysis are not suitable for the analysis of differentiated kidney organoids after differentiation.

**Figure 1:**
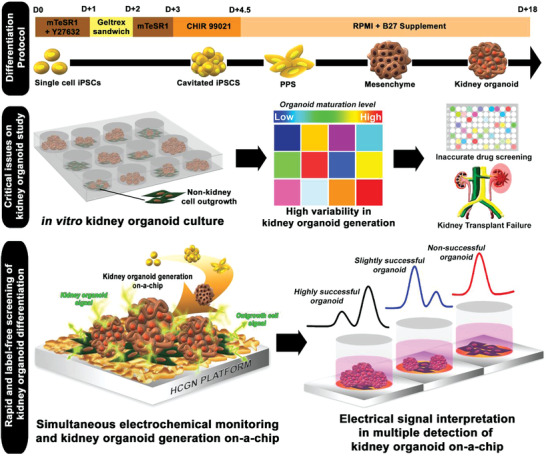
Schematic diagram representing kidney organoid on‐a‐chip and ECD method to monitor its successful, rapid, and label‐free generation.

This study addresses such issues by proposing an electrochemical method that effectively distinguishes immature and mature kidney organoids derived from human‐induced PSCs (hiPSCs), as shown in **Figure** **1**. Based on our previous research, highly proliferative cells (e.g., cancer cells and PSCs) produce unique redox signals at a specific potential of approximately 0 V (vs Ag/AgCl).^[^ ^44^, ^45^, ^46^ ^]^ Because the majority of off‐target cells also share similar characteristics (e.g., a rapid growth rate), we hypothesized that both undifferentiated hiPSCs at the early stage and off‐target cells generated during and after differentiation could be sensitively detected via the electrochemical method. Various extracellular matrix (ECM) components were tested by varying their coating conditions to identify a substrate that satisfies iPSC adhesion and only minimally increases the resistance of the electrode.

The electrical signals quantified from the iPSCs were used as an indicator of initial cell concentration prior to differentiation, which is essential for lowering the variations in organoid differentiation and maturation. Remarkably, similar to iPSCs, both the stromal cells (i.e., off‐target cells) and the kidney organoid‐specific cell types produce distinct electrochemical signals at different electrical potentials. The detection was repeated for multiple kidney organoids over the entire differentiation period (three weeks) to confirm the potential of these two different peaks as a key indicator of successful kidney organoid generation and maturation. Electrical signal intensities were then compared with the gene and protein expression levels of kidney‐specific markers (e.g., nephron progenitor cells, podocytes, proximal tubule cells, and vascular endothelial cells). These data suggest that the electrochemical method enables the non‐destructive and label‐free detection of kidney organoid maturation and off‐target cell generation. Furthermore, this approach can be applied to other types of organoids and used as a tool for organoid‐based drug screening, toxicity assessment, and therapeutics.

## 2 Results and Discussion

### 2.1 Optimization of ECM Materials on a Conductive Platform for Electrochemical Detection (ECD) Before In Vitro Kidney Organogenesis

PSCs possess extreme plasticity and are highly sensitive to the ECM, a structural support and biological reservoir for growth and differentiation factors.^[^ ^47^, ^48^ ^]^ Accordingly, different types of commercially available ECM materials (e.g., Matrigel, Geltrex, and Laminin‐521) were applied to conductive substrates with varying concentrations to identify the optimal ECM material for hiPSCs, which are the source for the generation of kidney organoids in vitro.^[^ ^49^ ^]^ In this study, a highly conductive gold nanostructure (HCGN) modified on transparent indium tin oxide (ITO)‐coated glass was fabricated and used as a primary platform for the electrical monitoring of hiPSC growth (Figure S1, Supporting Information). Figure S2, Supporting Information, illustrates the electrical characterization of the live cells using our conductive platform.

**Figure 2:**
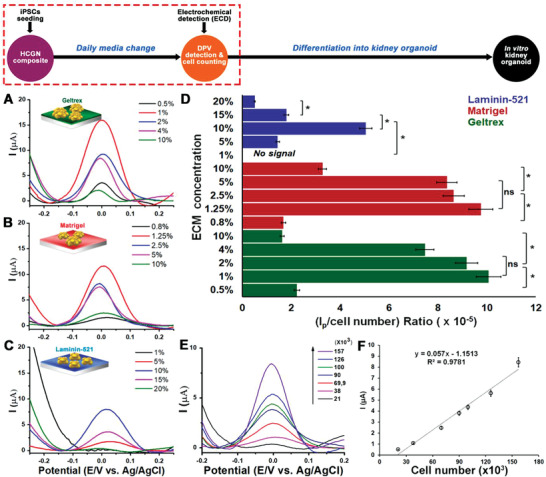
Optimization of ECM coating for hiPSCs cultivation on the HCGN platform. DPV signals of hiPSCs grown on various concentrations of A) Geltrex‐, B) Matrigel‐, and C) Laminin‐521. D) *I*_p_ values per cell number based on the data presented in A–(C). E) DPV signals with varying iPSC numbers. F) Linear correlations (*R* ^2^) of the calculated *I*_p_ value from (E) and the number of hiPSCs (One‐way ANOVA, *n* = 3, *p < 0.05).

First, the signal intensities obtained using different ECM materials modified on the HCGN platform were determined via differential pulse voltammetry (DPV) and then compared. The ECM materials included (1) Geltrex (0.5%, 1%, 2%, 4%, and 10%), (2) Matrigel (0.8%, 1.25%, 2.5%, 5%, and 10%), and (3) Laminin‐521 (1%, 5%, 10%, 15%, and 20%). As depicted in **Figure** **2**A–C, the electrical signal intensities per cell (*I*_p_ value/total cell number) were highest at the 1%, 1.25%, and 10% concentrations for Geltrex (10.1 × 10^–^^5^ µA), Matrigel (9.7 × 10^–^^5^ µA), and Laminin‐521 (5 × 10^–^^5^ µA), respectively. Exceeding the optimized concentrations of ECM materials resulted in decreased signal intensities due to the inhibition of cell attachment and proliferation as visualized by cell colony morphology, in addition to increased electrical resistance between the cell membrane and the electrode surface (Figure S3 and S4, Supporting Information). Given the performance of each ECM material in terms of both signal intensity and cell growth, the 1% Geltrex was chosen for kidney organoid on‐a‐chip generation because it can maintain both stem cell growth and multipotency with the ability to receive redox signals from living cells. Moreover, the hiPSCs grown on HCGN substrate were successfully maintained long‐term (≤7 days) in culture, thus demonstrating the good biocompatibility of our proposed conductive platform (Figure S5, Supporting Information).

Next, we sought to characterize the correlation between signal intensity and cell number. Cell confluency or concentration affects cell‐to‐cell interactions and the cellular uptake of differentiation factors, and is thus critical for the initiation of early differentiation.^[^ ^50^, ^51^ ^]^ Therefore, confirming the initial cell number without harming the cell‐to‐cell network is essential for inducing kidney organoid generation.

As illustrated in Figure 2E, the DPV signals increased with an increase in hiPSCs ranging from 21000 to 157000 cells with a correlation coefficient (*R* ^2^) of 0.9781 (Figure 2F). The limit of detection of the developed platform was 21363 cells. Reflecting on this result, the number of hiPSCs was successfully quantified only based on the electrical signals, and the applied electrical force did not harm the pluripotency of stem cells due to the short detection time (<10 s) and low potential range (−0.4 < *V* < 0.4, vs Ag/AgCl). Neither cell dissociation nor the addition of chemical agents was required to detect exact cell numbers before organoid generation. Thus, the developed conductive culture platform could be coupled with the ECD technique to precisely maintain the initial cell amount for all experimental groups used for kidney organoid generation, regardless of their growth rate differences.

### 2.2 Kidney Organoid Generation from hiPSCs on the Conductive Platform

**Figure 3:**
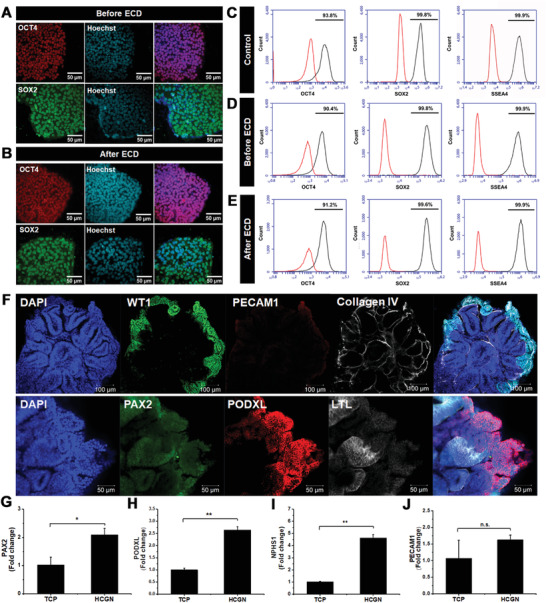
Proof‐of‐concept electrochemical assessment as a non‐destructive approach during kidney organoid generation. Representative immunofluorescence images of pluripotency markers [Oct4 (Red) and Sox2 (green)] and nuclei staining [Hoechst (blue)] for hiPSCs grown on a chip A) before and B) after ECD. Flow cytometry analysis of Sox2, Oct4, and SSEA‐4 expression of hiPSCs grown on TCP C) and the HCGN platform D–E). The red and black lines indicate the negative and positive cells populations, respectively. F) Confocal images of the expression of kidney organoid‐related markers (WT1, PECAM1, Collagen4, PAX2, PODXL, and LTL), indicating the biocompatibility of the HCGN platform toward kidney organoid formation. G–J) qPCR analysis of kidney organoid growth and maturation on HCGN platform (Student's *t*‐test, *n* = 3, *p < 0.05, ** *p* < 0.01).

After confirming the effectiveness of the electrochemical method as a nondestructive tool to detect the number of hPSCs before differentiation, the potential side effects of an electrical force applied to the cells was confirmed. As depicted in **Figure** **3**A,B, representative pluripotency markers including Oct4 and Sox2 were well‐expressed in the immuno‐stained hiPSCs subjected to the electrochemical measurements. No significant changes in cellular morphology were observed from the field emission scanning electron microscopy (FE‐SEM) images (Figure S6, Supporting Information). Flow cytometry results also revealed that the PSC markers (e.g., Sox2, Oct4, and SSEA‐4) were highly expressed in the hiPSCs regardless of the application of the electrochemical signals to conduct the measurements. Therefore, we concluded that a mild electrical force (|*V*| _vs Ag/AgCl_ ≤ 0.2) and short detection time (≤30 s) do not harm the pluripotency of stem cells when compared with hiPSCs grown on a typical tissue culture plate (TCP) (Figure 3C–E).

Next, the capability of the conductive platform (i.e., HCGN) to support direct self‐organization of hiPSCs and further kidney organoid generation was analyzed. As depicted in Figure 3F, both morphological characteristics and the marker expressions indicated the successful conversion of hiPSCs into kidney organoids. Distinct nephron‐like tubular structures appeared on the nucleus‐stained (4’,6‐diamidino‐2‐phenylindole, DAPI) images, a critical indicator of kidney organoid generation. Moreover, the segmented components expressed several representative kidney markers, including nephron progenitors (PAX2^+^), proximal tubules (LTL^+^), and glomeruli transcription factors (WT1^+^ and PODXL^+^). Both PECAM1 and Collagen IV, which are indicators of organoid vascularization and extracellular basement membrane deposition for the tubular structure, respectively, were also found to be highly expressed.

These findings confirm the successful generation of structural support and further vascularization of organoids. The mRNA expression levels of *PAX2*, *PODXL*, *NPHS1*, and *PECAM1* were analyzed to further confirm their successful differentiation on the conductive platform. Remarkably, the expression levels of all four distinct representative markers of kidney organoids were significantly enhanced on the HCGN platform (Figure 3G–J, Figure S7, Supporting Information). Additional studies are required to confirm our findings. However, we presumed that the unique nano‐topographical structure of HCGN provides low adhesion, resulting in the spontaneous cellular aggregation of iPSCs and enhanced differentiation.^[^ ^46^ ^]^ Therefore, we concluded that the fabricated conductive platform is suitable for the electrochemical label‐free monitoring of hiPSC growth and the in‐situ differentiation of stem cells for kidney organoid generation.

### 2.3 Non‐Destructive Electrical Method to Assess Kidney Organoid Generation

The existence of off‐target cells after differentiation and their cellular outgrowth are critical obstacles for the use of kidney organoids for both in vivo kidney regeneration and in vitro drug screening.^[^ ^20^, ^38^, ^52^, ^53^, ^54^ ^]^ After confirming the feasibility of the conductive platform as a substrate for kidney organoid generation, we applied the electrochemical method for the following purposes: 1) the detection and quantification of off‐target cells after differentiation and 2) the assessment of kidney organoid maturation. Conventional immunostaining and RT‐qPCR analysis were also performed with ECD to confirm the level of kidney organoid maturation and off‐target cell generation up to D24 over a three‐day time interval.

**Figure 4:**
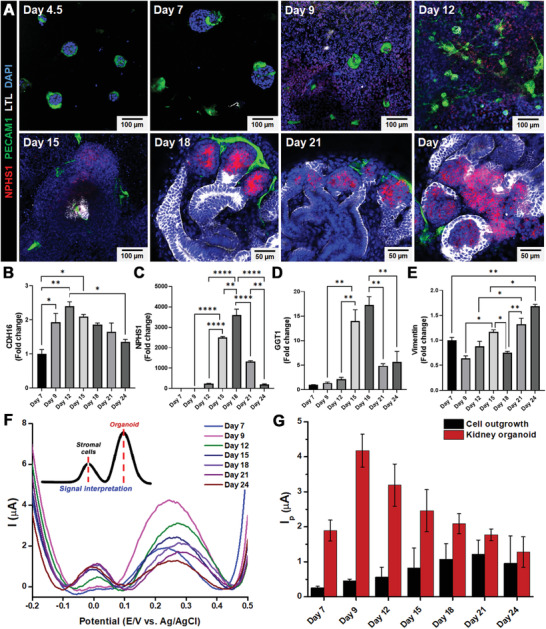
Real‐time electrochemical monitoring of successful kidney organoid generation on the HCGN platform. A) Representative time‐dependent confocal images of podocytes (NPHS1), proximal tubules (LTL), and vascular networks (PECAM1) during kidney organoid formation. Gene expression analyses of B) proximal tubules (CDH16), C) podocytes (NPHS1), D) renal proximal tubules (GGT1), and E) stromal cells (Vimentin). F) DPV voltammogram of kidney organoid formation from the early stage (day 7) to the late stage (day 24).G) The peak intensities observed in F) are presented as a bar graph. (One‐way ANOVA, *n* = 3, * *p* < 0.05, ** *p* < 0.01, *** *p* < 0.001, **** *p* < 0.0001).

On D18, the NPHS1 and LTL (i.e., the major markers of podocytes and proximal tubules, respectively) were highly expressed (**Figure** **4**A and Figures S8 and S9, Supporting Information). The vascularization of the organoid was also observed on D12 (PECAM^+^, green). Furthermore, several genes were upregulated on D12, including a proximal tubule early transcription factor (*CDH16*), as well as *NPHS1* and *GGT1*, which are podocyte and renal cell markers, respectively (Figure 4B–D, Figure S10, Supporting Information). Our findings indicated that the organoids were generated on D12 and reached their maturity on D18. However, the number of stromal cells (off‐target cells) also significantly affected the organoid maturation and further degradation, as confirmed by the mRNA expression level of vimentin, a marker of epithelial‐to‐mesenchymal transition (EMT) during organogenesis (Figure 4E).

Interestingly, when characterizing the DPV signals, two separate peaks appeared on the voltammogram at different electrical potentials (Figure 4F). Additionally, the kidney organoids grown on the ITO chip were used as a control group. As expected, the organoids did not emit any distinct electrical signal due to their low conductivity compared to the HCGN surface (Figure S11, Supporting Information). We first focused on the peak at 0.3 V (*I*_p_^K^ vs Ag/AgCl) because such potential differs entirely from what a homogeneous cell population generally produces at approximately 0 V. The intact kidney organoids (IKO) were separately detected from the cell outgrowth to determine the *I*_p_^K^ intensity originating from kidney‐specific cells. Interestingly, a single organoid peak current was observed at ≈0.2 V, which was slightly shifted compared to an actual *I*_p_^K^ potential at 0.3 V in a real‐time monitoring experiment (Figure S12A,B, Supporting Information). This might be due to the existence of off‐target cells among the successfully differentiated kidney organoids in the real‐time differentiation samples, thereby shifting the *I*_p_^K^ potential from its original value. This assumption was further proven by analyzing the dissociated kidney organoid (DKO), where the off‐target cell populations were mostly removed. The DPV peak of the DKO consistently appeared at 0.2 V, which coincided with the IKO signal. Furthermore, the qPCR data indicated a significant decrease in the expression of off‐target cell markers in DKO samples (Figure S12C, Supporting Information). This evidence indicated that the *I*_p_^K^ value was strongly associated with mature kidney organoids and could thus be used as a key parameter for DPV‐based monitoring of successful kidney organoid generation.

A comparison of the intensities of DPV signals with all representative kidney cell markers confirmed that the mRNA expression levels of CDH16 increased 2.3 times from D7 to D9, whereas the DPV signals increased approximately 2.2 times from D7 to D12 (Figure 4G). Despite variations in the time at which CDH16 expression level and electrical signal intensity (*I*_p_^K^) reached their peak, both parameters followed similar trends and continuously decreased with further differentiation until D24, when the degradation of tubular structures occurred. These phenomena indicated that the trends (both increasing and decreasing) of the electrical signal at 0.3 V (vs Ag/AgCl) and CDH16 gene expression levels were highly correlated with the generation of proximal tubule cells and can thus be used as an effective indicator of successful kidney organoid differentiation.

### 2.4 Electrochemical Monitoring of Nonsuccessful Kidney Organoid Generation

**Figure 5:**
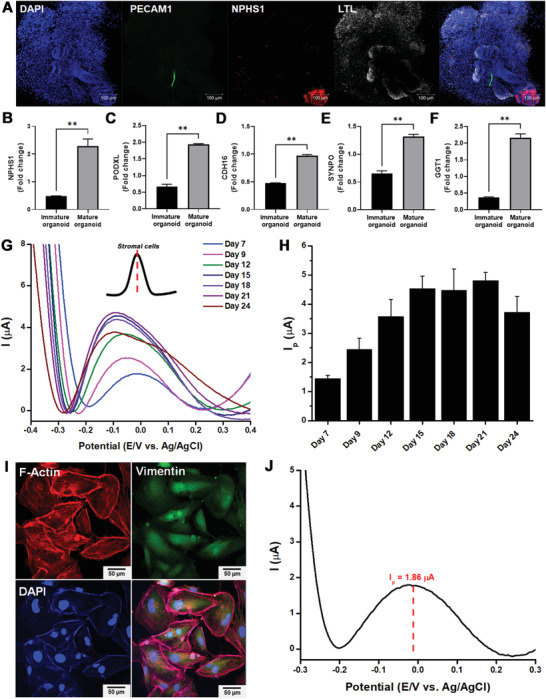
Real‐time electrochemical monitoring of nonsuccessful hiPSCs differentiation into kidney organoids. A) Representative confocal images of podocytes (NPHS1), proximal tubules (LTL), and vascular networks (PECAM1) in 16 days of kidney organoid formation. qPCR analyses of B) NPHS1, C) PODXL, D) CDH16, I SYNPO, and F) GGT1 gene markers expressed in kidney organoids. H) DPV voltammogram of kidney organoid formation from the early stage (day 7) to the late stage (day 24). H) The peak intensities observed in G) are presented as a bar graph. I) Fluorescence images and J) DPV detection of the outgrowth of stromal cells (Student's *t*‐test, *n* = 3, ** *p* < 0.01).

We also found that peaks at 0 V (*I*_p_^O^ vs Ag/AgCl) increased continuously from 0.25 µA on D7 to 1.22 µA on D21, which was consistent with the increase in stromal cell marker expression (vimentin). Based on this evidence, we sought to detect electrical signals from the kidney organoids lacking major cell types and tubular structures to support our hypothesis that electrical signals would appear at 0.3 and 0 V if the kidney organoids had reached maturity (e.g., tubule formation) or if the off‐target cells were present, respectively (**Figure** **5** and Figure S13, Supporting Information). As depicted in Figure 5A, tubular structures failed to form, as indicated by the low LTL^+^ expression, whereas the podocyte population (NPHS1^+^) was low in the stained images.

These phenomena resulted in the failure of organoid vascularization with negligible PECAM^+^ expression. The non‐successful kidney organoid formation on the HCGN platform was categorized as “immature organoid,” whereas the control group with well‐defined tubular structures was categorized as “mature organoid.” Immature organoids did not exhibit any significant formation of podocytes (*NPHS1*, *SYNPO, PODXL*), proximal tubules (*CDH16*), or renal proximal tubules (*GGT1*). This was consistent with their transcription factor expression levels, which were significantly lower than those of the control (Figure 5B–F, Figure S14, Supporting Information). Remarkably, the *I*_p_^O^ values increased steadily up to 15 days of differentiation whereas no detectable *I*_p_^K^ signals were observed in all immature organoid samples. The cells were separated from the organoids and re‐seeded on the HCGN platform for ECD to support our hypothesis that *I*_p_^O^ signals originate from off‐target cells (i.e., stromal cells).

As depicted in Figure 5J, clear voltammetric signals appeared at the same electrical potential, 0 V (vs Ag/AgCl), from the stromal cells highly expressing their representative marker, vimentin (Figure 5I). Based on the electrical potentials, we concluded that electrochemical signals can serve as indicators of kidney organoid generation and maturation, as well as off‐target cell formation. Such signal intensities can then be used for the nondestructive assessment of successful and nonsuccessful differentiation of iPSCs into kidney organoids. Therefore, they can be used as key indicators for organoid degradation, which generally occurs at the last stage of differentiation.

Next, we sought to determine if the culture media itself produced any electrical signals to rule out the possibility of false‐positive results. As illustrated in the DPV graph in Figure S15, Supporting Information, the media (mTeSR media for hiPSCs and RB media for kidney organoids) did not emit any measurable signals in any of the detection experiments. The conditioned RB media was also assessed to determine if there were any detectable signals from cell secretion compounds or metabolites released in the culture medium. No meaningful signals were detected, which confirmed that the *I*_p_^K^ and *I*_p_^O^ peaks were solely generated from kidney organoids and stromal cells, respectively.

We further investigated the specificity of our proposed system for kidney organoid detection by confirming that the *I*_p_^K^ signals were not systematic errors. To assess this, human cancer cells and normal vascular cells (HUVEC) were detected through DPV. Together with the previous characterization of culture medium in Figure S15, Supporting Information, our findings confirmed that both culture medium only and normal vascular cells emitted no meaningful signals, whereas a clear DPV peak appeared at 0 V in cancer cells, similar to the undifferentiated iPSCs (Figure S16, Supporting Information). This finding confirmed that the signals at 0.3 V did not originate from non‐kidney cells or rapidly growing cells (e.g., cancer cells and PSCs).

As discussed earlier, our findings indicated that the matured kidney organoids with distinct tubular structures emitted a strong electrochemical signal at 0.3 V even after the cell dissociation process (Figure S12, Supporting Information). In this case, the relative peak intensities at 0.3 V became more apparent due to the partial elimination of off‐target cells during the dissociation process. Collectively, our findings partially demonstrated that *I*_p_^K^ values can serve as indicators of the maturity of kidney organoids. However, additional studies are needed to gain further insights into the interpretation of *I*_p_^K^ values, which could be used as a reliable parameter for successful kidney organoid differentiation.

## 3 Conclusion

Our study proposed a new technique for the rapid and label‐free assessment of kidney organoid generation in vitro. Laboratory‐scale kidney organoid culture has thus far been limited by the lack of effective methods to study the immaturity and variability of differentiated organoids and the detection of outgrowing off‐target cells without harming the distinct structure of the organoids. An electrochemical method capable of monitoring the process of kidney organoid generation from iPSCs is highly promising because it can eliminate destructive pre‐processing steps, including cell lysis, dissociation, and fixation.

The 1% Geltrex‐modified HCGN platform was proven to support the hiPSC growth and pluripotency without hindering the detection of their redox signal. Prior to inducing the kidney organoid differentiation, the number of undifferentiated hiPSCs was successfully quantified based solely on electrical signals. The detected hiPSCs were successfully induced into kidney organoids, which was confirmed by immunohistochemical staining of several markers for kidney‐specific cell types (PAX2^+^/LTL^+^/WT1^+^/PODXL^+^) and the microscopic observation of a distinct tubular structure, along with the increase of mRNA expression levels of kidney‐specific genes (*PAX2*, *PODXL*, *NPHS1*, and *PECAM1*) when compared with conventional TCPs.

Remarkably, two peaks were detected at ≈0 V (*I*_p_^O^) and ≈0.3 V (*I*_p_^K^) in the voltammogram while monitoring kidney organoid generation. The first peak, *I*_p_^O^, corresponds to the off‐target cell outgrowth, whereas the *I*_p_^K^ values are specific to the kidney organoid generation. Strong *I*_p_^K^ signals were observed in organoids with a distinct tubular structure. Such signals were highest on D7 and decreased steadily until D24. The *I*_p_^O^ values continuously increased from D7 to D24, in contrast to the *I*_p_^K^ signal intensities.

The *I*_p_^K^ signal intensity trend was consistent with the expression levels of several kidney‐specific cell types, particularly the proximal tubule early transcription factor (CDH16). No *I*_p_^K^ signals were observable from the immature kidney organoid that failed to exhibit a tubular structure but produced powerful *I*_p_^O^ signals representing off‐target cell outgrowth. The *I*_p_^O^ values were further matched with the signals of the separated stromal cells (i.e., off‐target cells), thus supporting our hypothesis that the *I*_p_^O^ and *I*_p_^K^ values were indicative of off‐target cell outgrowth and successful kidney organoid generation, respectively.

As indicated in our electrical circuit diagram and all DPV signals, active cellular redox molecules play a critical role in electrical signal generation at a specific potential and eventually affect the contact resistance (*R*_gap_) outcome. At this point, the existing *R*_gap_ value representing the cell‐electrode interface becomes negligible; however, the mechanisms that govern this phenomenon remain unclear. Thus, future studies should focus on the correlation between cell adhesion and the *R*_gap_ effects (e.g., performing single‐cell RNA sequencing to assess cell adhesion‐related gene markers).

**Figure 6:**
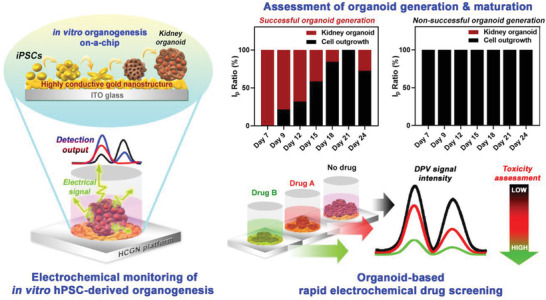
Summary and potential applications of the HCGN platform for organoid‐based rapid drug screening.

The generation of organoids through stem cell differentiation involves complex biochemical processes, resulting in outcome variations. This phenomenon is not limited to kidney organoid generation, as other types of organoids (e.g., intestine, lung, liver, and brain) also originate from PSCs and undergo complex developmental processes. Therefore, precise characterizations and analysis of the generated organoids concerning their structural and compositional maturation are crucial to promoting their therapeutic use, in vitro drug screening, and toxicity testing. Based on our preliminary study, the toxicity of several well‐known nephrotoxic drugs (e.g., cisplatin and nedaplatin) toward kidney organoids was successfully monitored using our proposed method (Figures S17 and S18, Supporting Information). Our findings demonstrated that the proposed approach enables the nondestructive and label‐free detection of kidney organoid maturation and off‐target cell generation, and is thus a promising tool in pharmacological research. Future studies should thus assess its practical applications (**Figure** **6**).

## 4 Experimental Section

### Materials

The materials included an indium tin oxide (ITO) glass substrate (U.I.D Korea), gold (III) chloride trihydrate (Sigma–Aldrich), poly(ethylene glycol) 200 (PEG, Sigma–Aldrich), polydimethylsiloxane (PDMS, Dow Corning Corp.), Dulbecco's modified eagle medium/nutrient mixture F–12 (DMEM/F–12, Gibco), minimum essential medium (MEM, Gibco), fetal bovine serum (FBS, Gibco), antibiotic‐antimycotic (Gibco), Triton X‐100 (Sigma), and Dulbecco's phosphate‐buffered saline (DPBS, Gibco). All reagents were of analytical grade. The solutions used in this study were prepared with deionized (DI) water that was purified using a Millipore Milli‐Q direct water purification system (EMD Millipore).

### Highly Conductive Gold Nanostructure (HCGN) Platform Fabrication

ITO glass substrates were subsequently cleaned with a 1% Triton X–100 solutions, DI water, and 70% ethanol in an ultrasonic bath. A plastic chamber was affixed onto the sterilized ITO glass using PDMS, a biofriendly glue, before the electrochemical deposition of the gold mixture solution to maintain cell growth on the chip. The gold mixture solution consisted of 5 mM gold (III) chloride solution with PEG at a 50:1 ratio. The HCGN fabrication on the substrate was performed using the multistep potential (MSP) channel on the electrochemical instrument at 120 s, as previously reported.^[^ ^45^ ^]^ The sterilized HCGN/ITO was coated with the optimal ECM material diluted in DMEM/F12 at the desired ratio to generate the HCGN composite. It was then placed in an incubator (37 °C, 5% CO_2_) for 1 h (Geltrex), 1.5 h (Matrigel), and 2 h (laminin‐521). Finally, the ECM‐coated solution was discarded before hiPSC seeding.

### Cell Culture

The hiPSC CMC11 and WTC11 cell lines were obtained from the Catholic University and cultivated on a six‐well TCP coated with 1% Geltrex LDEV‐free reduced‐growth‐factor basement membrane matrix without phenol red (GelTrex, Gibco, #A1413202) diluted in DMEM/F12 media. The mTeSR1 media (Stem Cell Technologies, #0 5850) was used for the hiPSC culture. Before cell plating, the pellet was washed with mTeSR1 media containing 10 µM Y‐27632 (Rho‐kinases [ROCK]) inhibitor (Tocris). Following the HCGN platform fabrication in the previous step, the cultivated hiPSCs were dissociated into cell clusters using ReLeSR (Stem cell technologies) to seed the cells onto the chip surface. The mTeSR1 media were changed daily up to the day of DPV detection, at which point the cells on the chip were analyzed using a cell counting kit (CCK‐8) to determine the viable cell number.

### Kidney Organoid Generation

The hiPSC CMC11 cell line was used between passages 30 and 35, whereas the WTC11 cell line was cultured between passages 50 and 60. Kidney organoid differentiation was performed as described previously.^[^ ^23^ ^]^ The hiPSCs were plated at a density of 5 × 10^3^ cells per well in 24‐well plates containing mTeSR1 medium (Stem Cell Technologies) with the addition of 10 µM ROCK inhibitors on glass plates (LabTek) coated with 3% GelTrex (day −3). At day −2, the medium was changed with 1.5% GelTrex in mTeSR1, followed by mTeSR1 (day −1), RPMI media (Thermo Fisher Scientific) + 12 µM CHIR99021 (Tocris) (day 0), and RPMI + B‐27 supplement (Thermo Fisher Scientific) (day 1.5). Next, the cells were fed every 2–3 days to induce kidney organoid differentiation. Organoids were transferred onto the GelTrex‐coated HCGN platform before the electrochemical assessment.

### Electrochemical Detection (ECD)

The DPV method was conducted using a DY2013 Potentiostat (EG Technology Inc.). A platinum wire was used as the counter electrode, whereas Ag/AgCl (1 M KCl) was used as the reference electrode and the HCGN platform as a working electrode. The mTeSR1 media was exchanged prior to the ECD to prevent any possible signal interferences from the redox molecules released by the hiPSCs during long‐term cultivation. The resulting scan rate, pulse amplitude, and pulse width were 100 mV s^−1^, 50 mV, and 20 ms, respectively. All DPV detections were performed at room temperature (25 ° C). These conditions were applied to all experiments. The Ip values were determined by subtracting the baseline current from the current value of DPV at *E*p = 0 V.

### Immunofluorescence and Immunohistochemistry Analysis

The hiPSCs were fixed with 10% neutral buffered formalin (NBF) solution and permeabilized with 0.3% Triton X–100. The cells were then incubated with primary antibodies: Oct4 (1:200, Abcam) and Sox2 (1:100, cell signaling) for 1 h at room temperature. Afterward, the cells were washed with DPBS and further incubated with either goat anti‐rabbit IgG (H+L) with a highly cross‐adsorbed secondary antibody (Alexa Fluor 594, Invitrogen) for visualizing Oct4 or donkey anti‐mouse IgG Northern Lights NL557‐conjugated antibody to visualize Sox2 (R&D Systems). Hoechst 33 342 (Sigma–Aldrich) was used to counterstain the nuclei. All fluorescence images were visualized using an Eclipse 80i fluorescence microscope (Nikon, Japan).

For immunofluorescence analysis of kidney organoids, the samples were fixed on day 18 unless otherwise indicated. An equal volume of phosphate‐buffered saline (PBS) (Thermo Fisher Scientific) + 8% paraformaldehyde (Electron Microscopy Sciences) was added to the medium for 10 min before the fixation procedure, after which the samples were washed three times with PBS. The fixed organoids were blocked with 5% donkey serum (Millipore) + 0.3% Triton X‐100 in PBS, followed by overnight incubation using 3% bovine serum albumin (BSA, Sigma) in PBS containing primary antibodies. The cells were then washed and incubated with Alexa Fluor secondary antibodies (Invitrogen) for 1 h at room temperature. The nucleus part was counterstained with DAPI. All primary and secondary antibodies are listed in Table S1, Supporting Information.

### Scanning Electron Microscopy (SEM) Analysis

The hiPSCs were fixed with 10% NBF solution, followed by dehydration steps using a series of ethanol concentrations in distilled water: 50%, 60%, 70%, 80%, 90%, and 100% of ethanol (EtOH). Next, the dehydrated samples were chemically dried with hexamethyldisilazane (HMDS) at three ratios with absolute ethanol. The EtOH:HMDS ratios were 1:2, 1:1, and 2:1, and the procedure was conducted every 20 min. The drying step was then finalized with pure HMDS for 20 min, followed by evaporation prior to SEM visualization.

### Quantitative Polymerase Chain Reaction (qPCR) Analysis

The RNA was extracted from cell lysates using the BioFACT Total RNA Prep Kit (BioFACT) and reverse transcribed with M‐MLV reverse transcriptase (TOYOBO) according to the manufacturer's instructions. The cDNA was mixed with SYBR Green (Enzynomics) and gene‐specific primers. All qPCR primers are listed in Table S2, Supporting Information. All the qPCR analyses were performed in triplicate, and the relative mRNA expression levels were determined using the 2^–ΔΔ^ *^Ct^* method.

### Flow Cytometry Analysis

A fluorescence‐activated cell sorting (FACS) analysis was performed using a human/mouse PSC multi‐color flow cytometry kit (R&D Systems, Catalog No. FMC001). The hiPSCs were harvested and washed with PBS. The pellet was immediately resuspended in a 10% NBF solution for the fixation step, followed by washing. Several pluripotency markers were checked using the kit component (Sox2, Oct‐3/4, SSEA‐4, and SSEA‐1). All FACS analyses were performed using the BD Accuri C6 Plus Flow Cytometer (BD Bioscience).

### Statistical Analyses

All data are presented as mean ± standard deviation (SD) with three replication samples. Pair‐wise comparisons were conducted using the unpaired Student's *t*‐test. Multiple comparisons were conducted through one‐way analysis of variance (ANOVA) coupled with Tukey's post hoc test. Significant differences were marked as *(*p* < 0.05) or **(*p* < 0.01).

## Conflict of Interest

The authors declare no conflict of interest.

## Supporting information

Supporting information

## Data Availability

The data that support the findings of this study are available from the corresponding author upon reasonable request.
