# Supplementary material for: In Situ Detection of Kidney Organoid Generation From Stem Cells Using a Simple Electrochemical Method
Source: Adv Sci (Weinh). 2022 May 4;9(20):2200074. doi: 10.1002/advs.202200074 (PMC9284177; doi:10.1002/advs.202200074)
Supplement: Supplementary file 1 — Supporting information [file ADVS-9-2200074-s001.pdf]

Supporting Information

In situ detection of kidney organoid generation from stem cells using a simple electrochemical method

Intan Rosalina Suhito, Jin-Won Kim, Kyeong-Mo Koo, Sun Ah Nam, Yong Kyun Kim, and Tae-Hyung Kim**

Table S1. Primary and secondary antibodies used this study

Antibody Name	Source	Ratio
Anti-Oct4	Abcam, ab19857	1:200
Sox2 Mouse mAb	Cell Signaling Technology, L1D6A2	1:100
Biotinylated Lotus Tetragonolobus Lectin (LTL)	Vector Labs, B-1325-2	1:150
Human Podocalyxin	R&D systems, AF1658	1:150
Anti-Claudin 1	Abcam, ab15098	1:150
Goat Anti-Type IV Collagen-UNLB	SouthernBiotech, 1340-01	1:150
Human Nephrin (NPHS1)	R&D systems, AF4269	1:150
Purified anti-Pax-2	Biolegend, 901001	1:150
Recombinant Anti-Wilms Tumor Protein (WT1)	Abcam, ab89901	1:150
Anti-CD31	Abcam, ab9498	1:150
Goat Anti-Rat IgG H&L (Alexa Fluor® 488)	Abcam, ab150157	1:200
Goat anti-Rabbit IgG (H+L) Highly Cross-Adsorbed Secondary Antibody, Alexa Fluor 594	Invitrogen, #A-11037	1:200
Cy™3 AffiniPure F(ab')□ Fragment Donkey Anti-Rabbit IgG (H+L)	Jackson ImmunoResearch Laboratories, 711-166-515	1:200
Donkey Anti-Sheep IgG Antibody, Cy3 conjugate, Species Adsorbed	MERCK Millipore, AP184C	1:200
Donkey Anti-Goat IgG H&L (Alexa Fluor® 647) preadsorbed	Abcam, ab150135	1:200
Streptavidin, (Cy5)	Invitrogen, SA1011	1:200
Donkey anti-Rabbit IgG (H+L) Highly Cross-Adsorbed Secondary Antibody, Alexa Fluor 488	Invitrogen, A-21206	1:200
Donkey anti-Mouse IgG (H+L) Highly Cross-Adsorbed Secondary Antibody, Alexa Fluor 488	Invitrogen, A-21202	1:200

Table S2. PCR primers used in the experiment

Gene	Primer sequence 5' to 3'
AQP1	Forward: GTCCAGGACAACGTGAAGGT<br>Reverse: GAGGAGGTGATGCCTGAGAG
ATP1A1	Forward: CCAATTGTGTTGAAGGCACC<br>Reverse: CCGTGATGATGTGGATAAAATGT
CDH16	Forward: CCTCATCCTCATTTTCACC<br>Reverse: GGGCTTCTACTCTGTCCTG
CLCNKA	Forward: CCCTCTACAAGACCAGTTTCCG<br>Reverse: CGCTGACAGAAGAGGTAAGCAC
ECAD	Forward: CGAGAGCTACACGTTCAGG<br>Reverse: GGGTGTCGAGGGAAAAATAGG
GATA3	Forward: CGTCCTGTGCGAACTGTCA<br>Reverse: GTCCCCATTGGCATTCCTCC
GADPH	Forward: AGGGCTGCTTTTAACTCTGGT<br>Reverse: CCCCACTTGATTTTGGAGGGA
GGT1	Forward: TGACCTTCAGGAGAACGAGA<br>Reverse: TCTTCTTCATGGCTCTGCGT
NPHS1	Forward: GGCTCCCAGCAGAAACTCTT<br>Reverse: CACAGACCAGCAACTGCCTA
PAX2	Forward: CCCAAAGTGGTGGACAAGAT<br>Reverse: GAAAGGCTGCTGAACTTTGG
PDGFR-b	Forward: TGCAGACATCGAGTCCTCCAAC<br>Reverse: GCTTAGCACTGGAGACTCGTTG
PECAM1	Forward: TCATTACGGTCACAATGACGA<br>Reverse: GAGTATCTGCTTTCCACGGC
PKD1	Forward: AACAAGTCTTTGGCCATCAC<br>Reverse: TACTCGTTCAGCACGGTGAC
PKD2	Forward: TCTTGCCAATTTCAGCCTTT<br>Reverse: GCACAACGATCACAACATCC
PODXL	Forward: GATAAGTGCGGCATACGGCT<br>Reverse: GCTCGTACACATCCTTGGCA,
SYNPO	Forward: GCTGAGGAGGTGAGATGCAG<br>Reverse: CTCTGGAGAAGGTGCTGGTG
VEGF-A	Forward: GTCCAACATCACCATGCAGATTA<br>Reverse: GCTGTAGGAAGCTCATCTCTC
WT1	Forward: GCGGAGCCCAATACAGAATA<br>Reverse: GATGCCGACCGTACAAGAGT
MAP2	Forward: CTCAGCACCGCTAACAGAGG<br>Reverse: CATTGGCGCTTCGGACAAG
PMEL	Forward: AGGTGCCTTTCTCCGTGAG<br>Reverse: AGCTTCAGCCAGATAGCCACT
MYLPF	Forward: GAAGGACAGTAGAGGGCGGAA<br>Reverse: TCTGGTCGATCACAGTGAAGG
MYOG	Forward: GGGGAAAACTACCTGCCTGTC<br>Reverse: AGGCGCTCGATGTACTGGAT

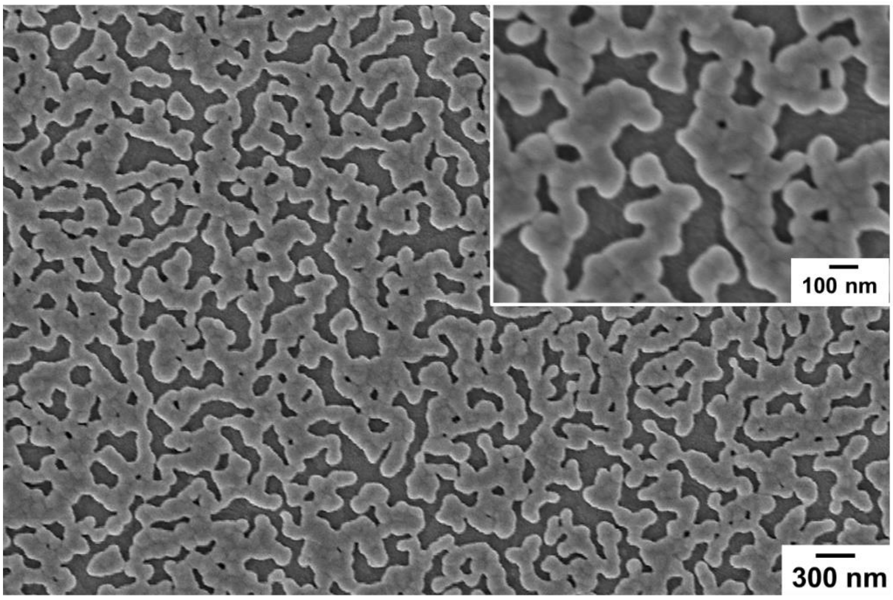

Figure S1. Scanning electron microscopy (SEM) image of HCGN substrate with low and high magnification (inset figure).

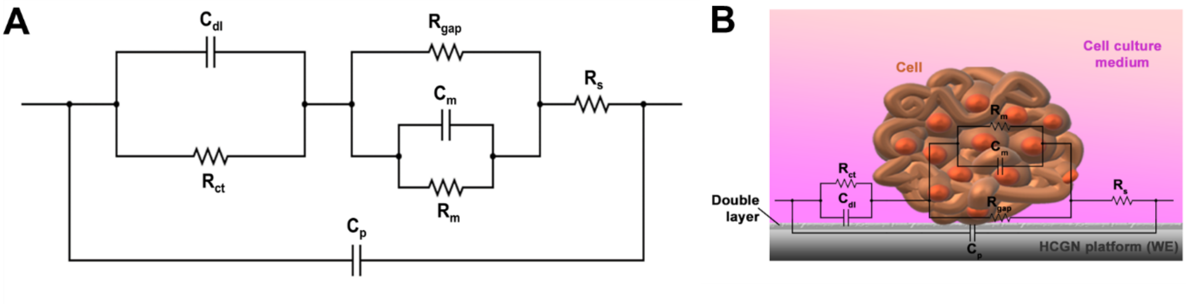

Figure S2. Electrical fundamentals of the cell-HCGN model. (A) Equivalent electrical circuit diagram and (B) schematic illustration of the circuit flow applied to the electrochemical detection components of our proposed system. C_{dl}: Capacitance of electrical double layer; C_m: Capacitance of cell membrane; R_s: Resistance of solution (cell medium); R_{ct}: Charge transfer resistance; R_{gap}: Resistance of cell-HCGN distance; R_m: Resistance of cell membrane, C_p: Parasitic capacitance.

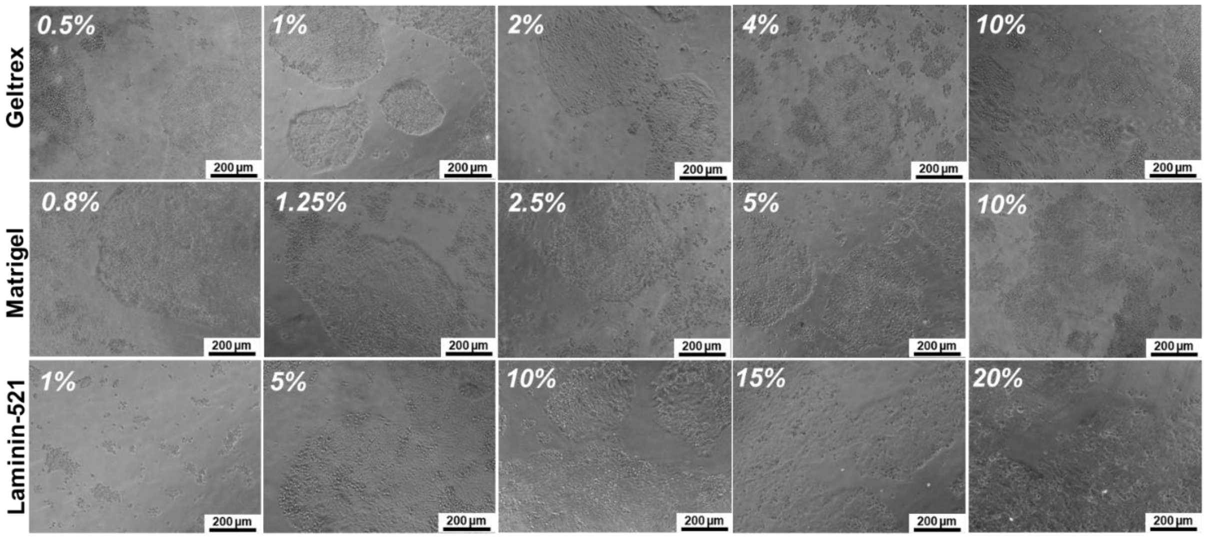

Figure S3. Optical microscopic images of hiPSCs grown on HCGN coated with several extracellular matrix (ECM) materials at various concentrations.

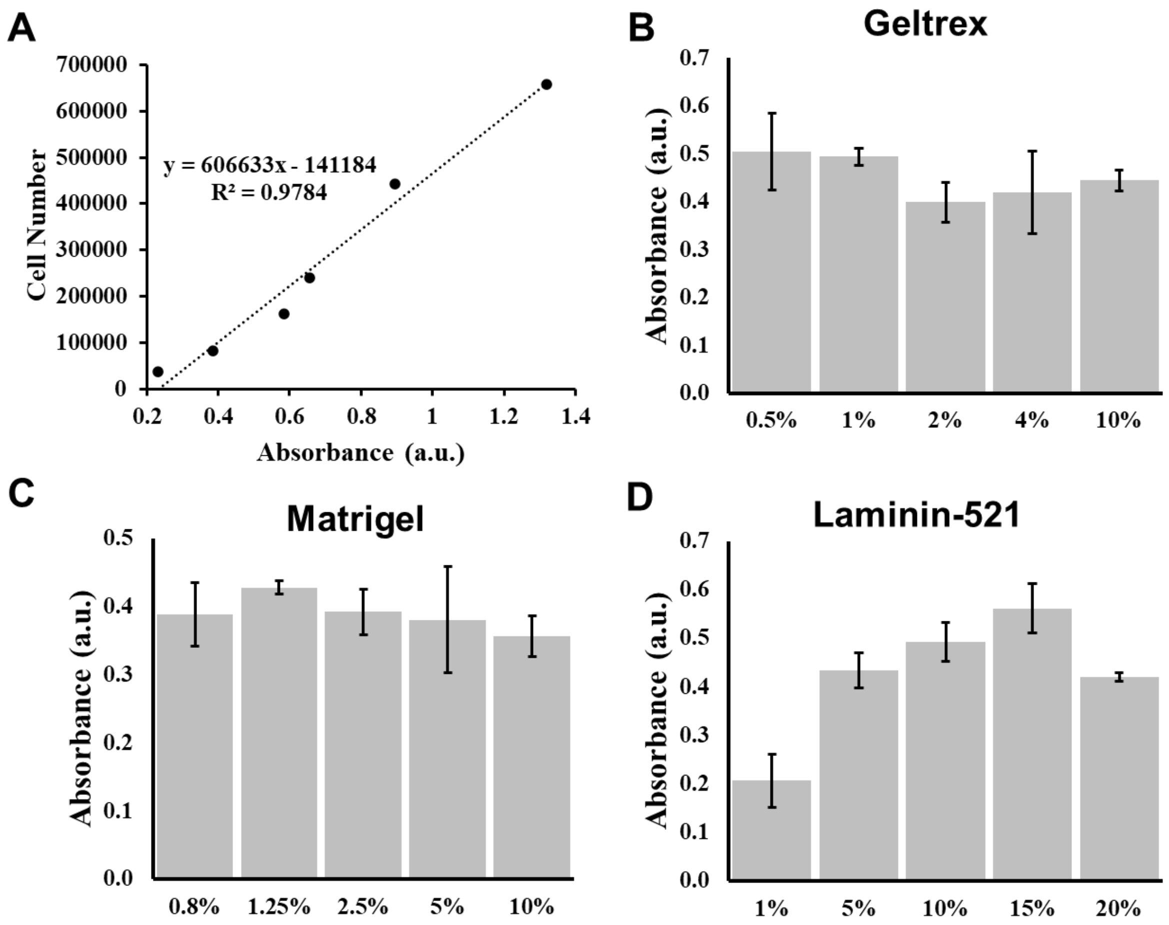

Figure S4. Determination of hiPSC numbers through CCK-8 assay. (A) Standard curve of CCK-8 method towards cell viability of hiPSCs. CCK-8 data of hiPSCs grown on various concentrations of (B) Geltrex-, (C) Matrigel-, and (D) Laminin-521-coated HCGN chip.

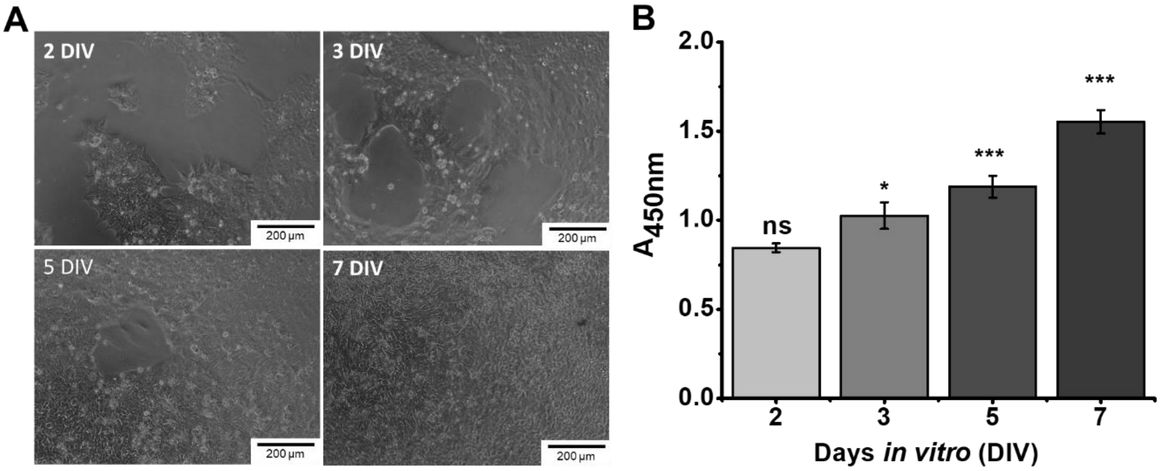

Figure S5. CCK-8 viability testing for long-term culture of hiPSCs on the HCGN platform. (A) optical microscopic images and (B) histogram showing the CCK-8 absorbance of hiPSC growth at 2, 3, 5, and 7 days *in vitro* (DIV) (Student's t-test, n = 3, $*p < 0.05$, $*** p < 0.001$, ns = not significant).

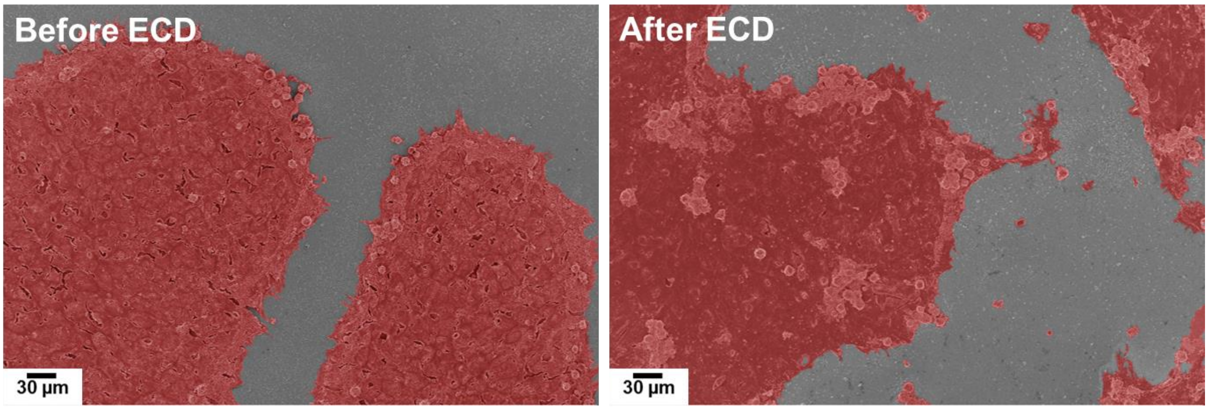

Figure S6. Pseudo-color scanning electron microscopic (SEM) images of hiPSC colony before and after ECD analysis.

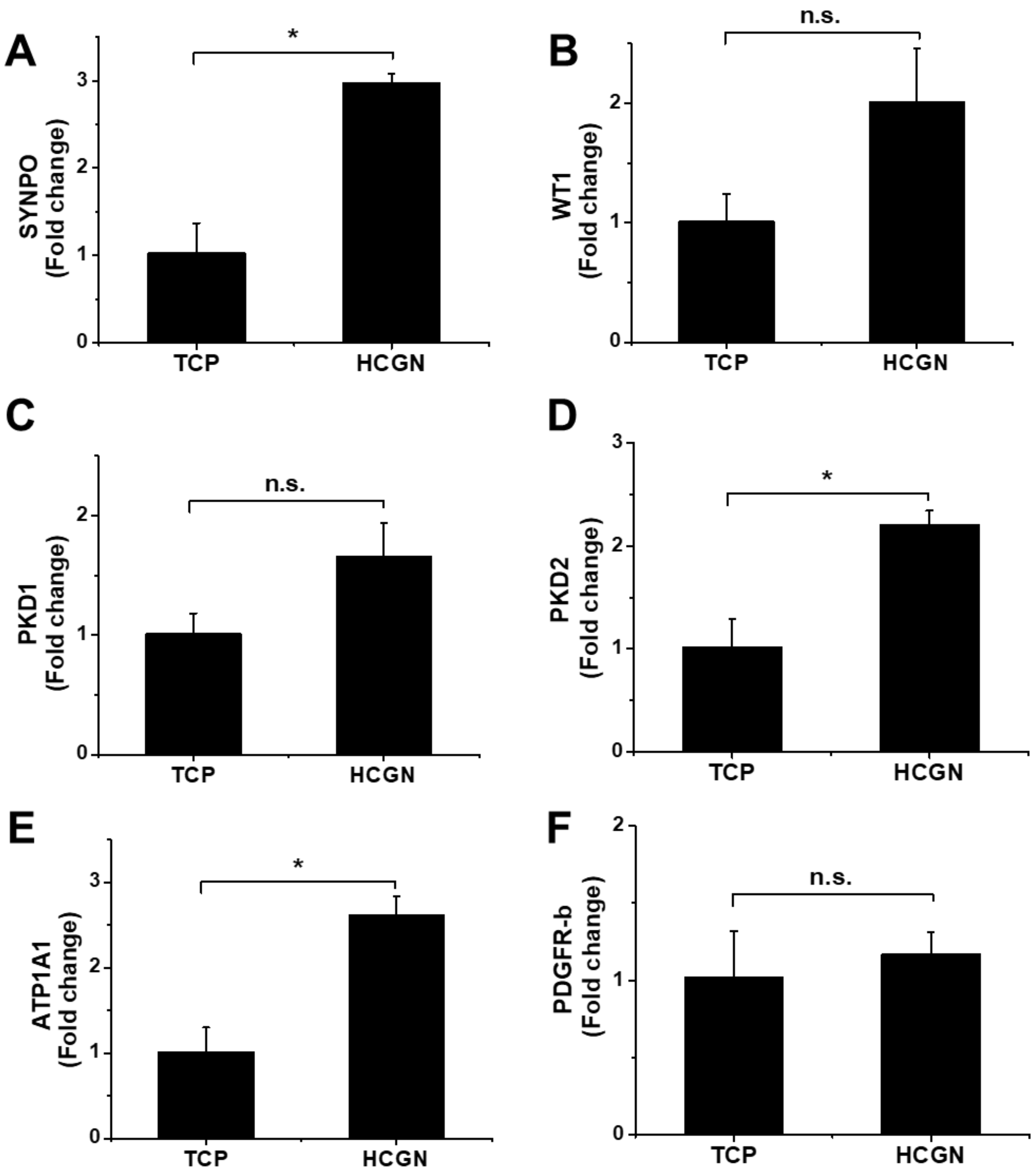

Figure S7. qPCR analysis of the genes related to kidney organoid growth and maturation on the HCGN platform. Expression of (A–B) podocytes markers, (C–D) maturation-associated markers in ciliary assembly for transient receptor potential channel, (E) a tubule epithelial transporter marker, and (F) a pericyte-like cell marker.

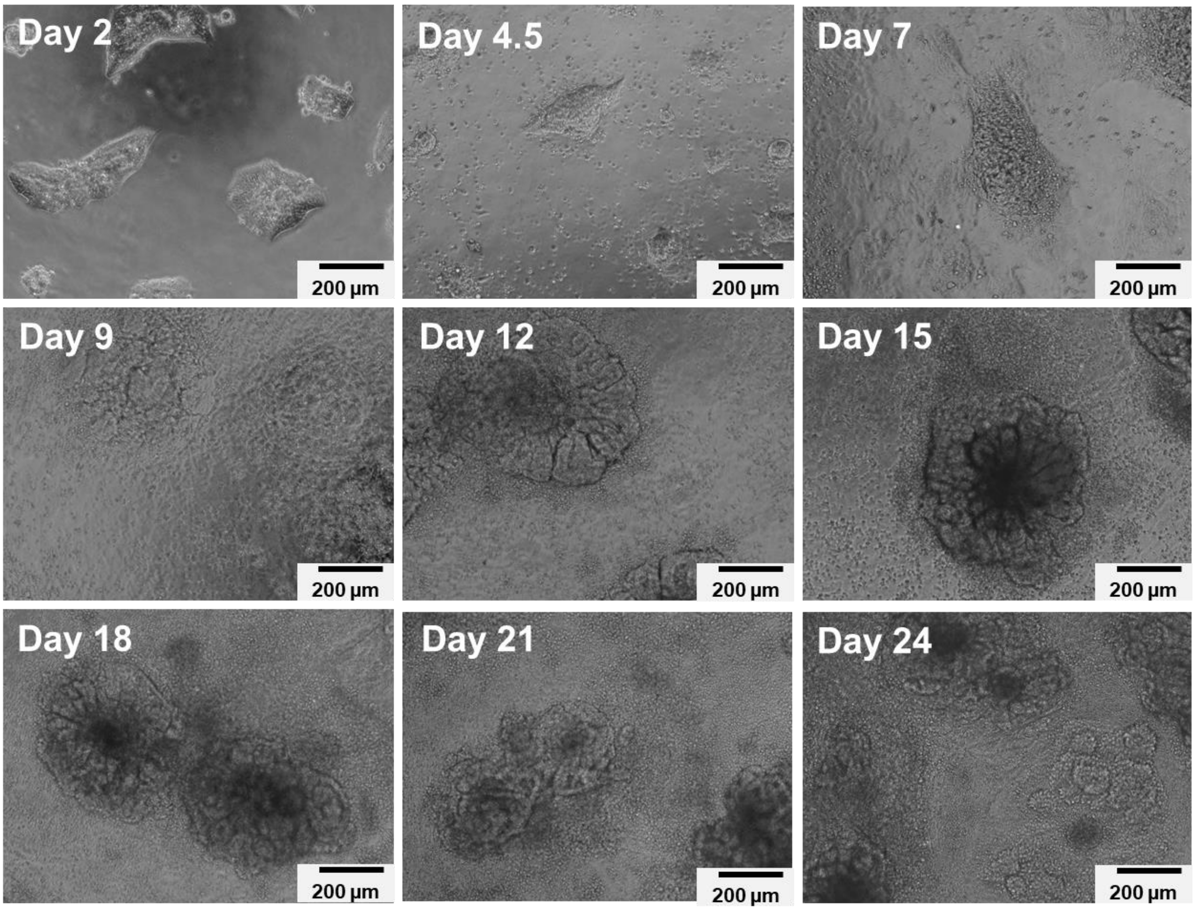

Figure S8. Time-dependent optical images of hiPSC differentiation into kidney organoid.

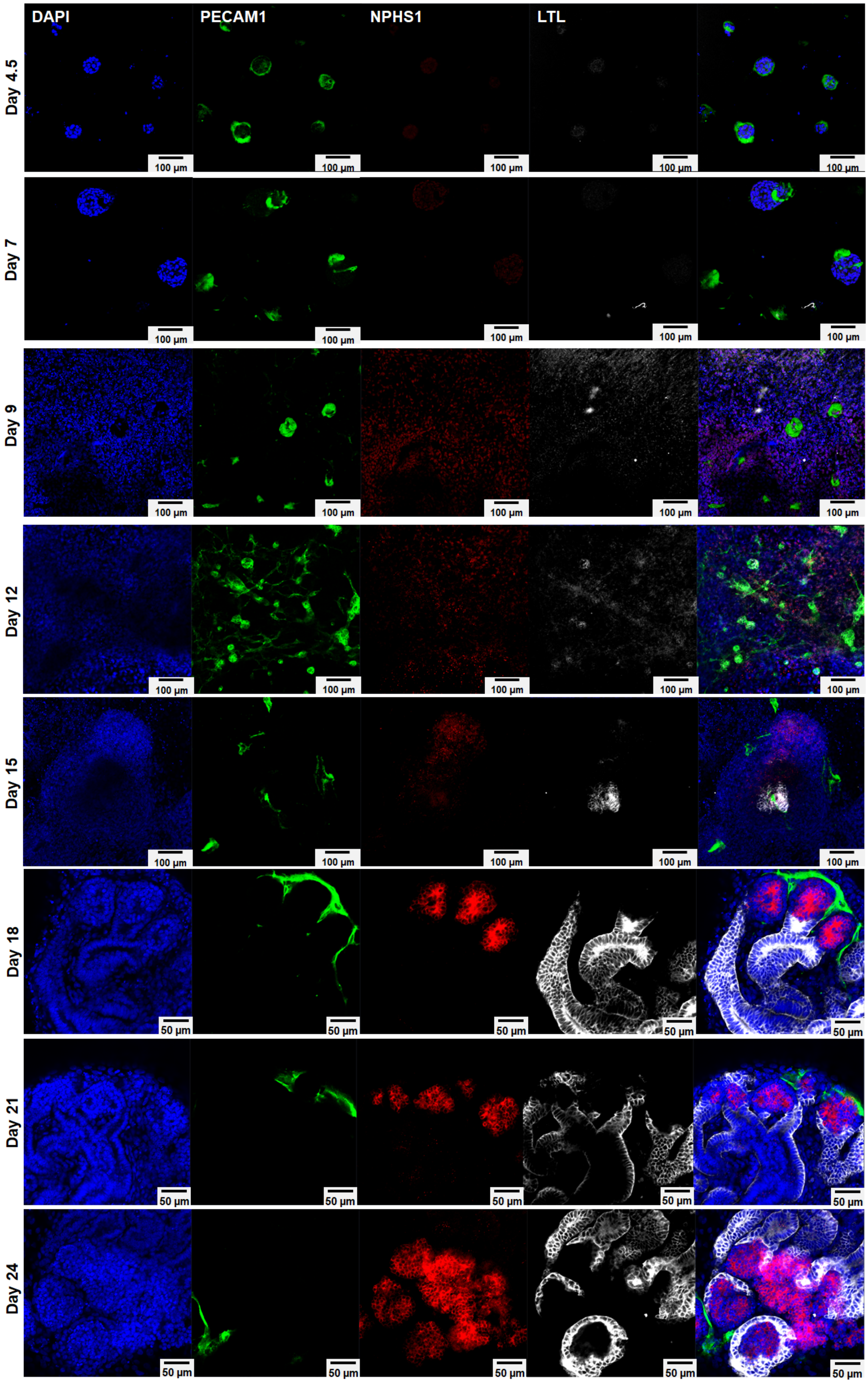
DAPI
PECAM1
NPHS1
LTL
Day 4.5
Day 7
Day 9
Day 12
Day 15
Day 18
Day 21
Day 24
100 µm
50 µm

Figure S9. Confocal images representing podocytes (NPHS1), proximal tubules (LTL), and vascular networks (PECAM1) during kidney organoid formation from the early (day 7) to late (day 24) stages.

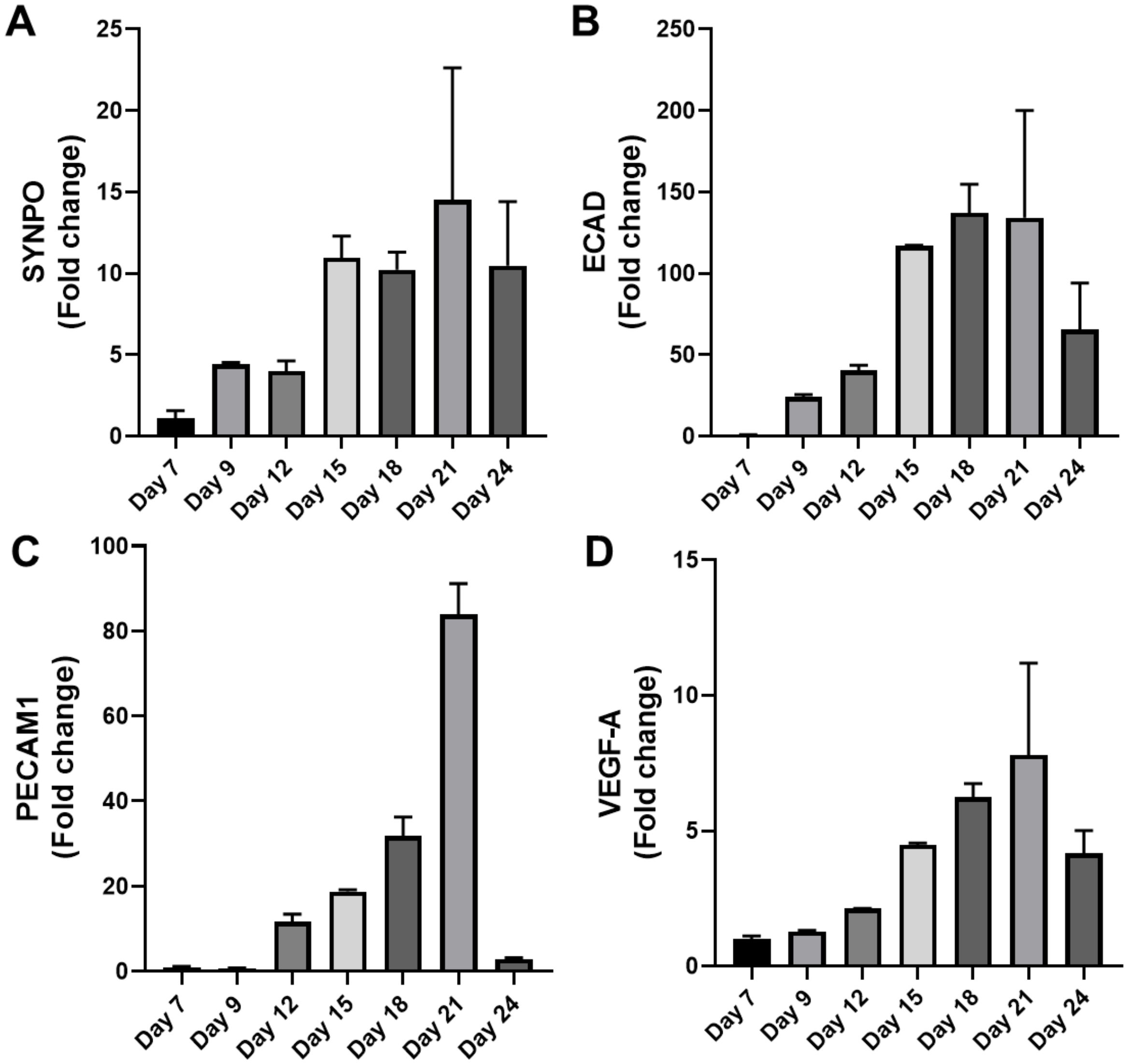

Figure S10. qPCR analyses of genes related to kidney organoid growth and maturation on the HCGN platform. Expression of (A) podocytes marker, (B) distal tubules marker, and (C–D) organoid vascularization-related markers.

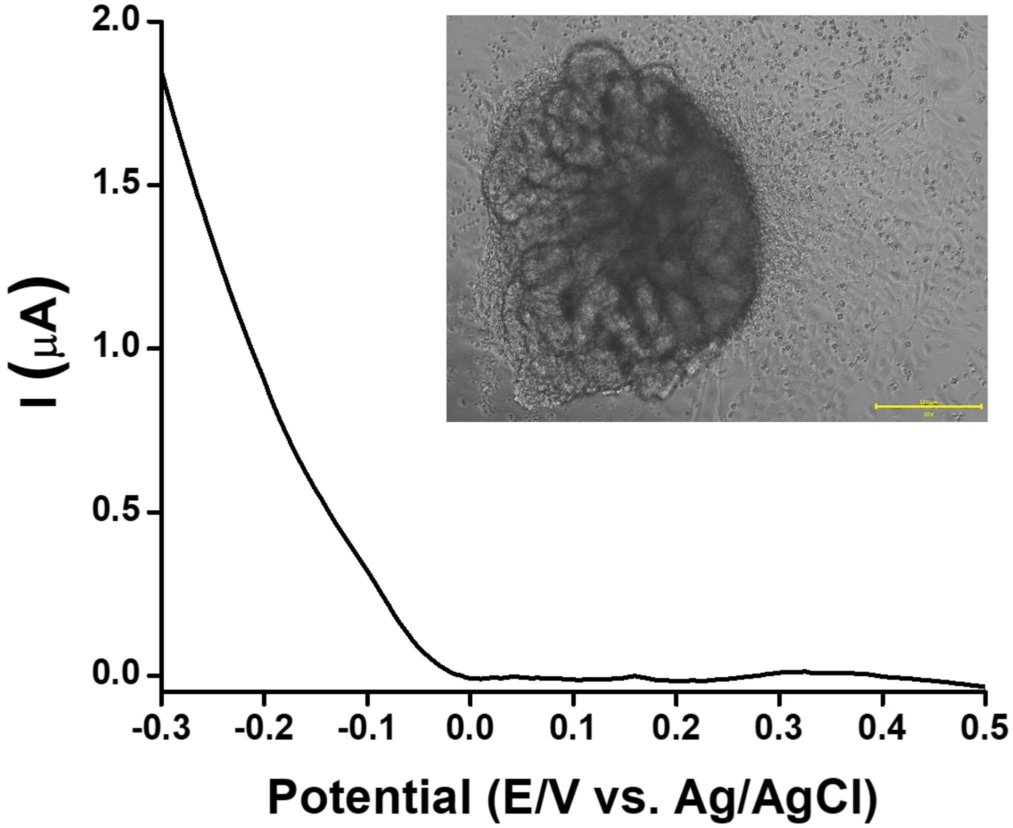

Figure S11. DPV detection of kidney organoid grown on ITO chip.

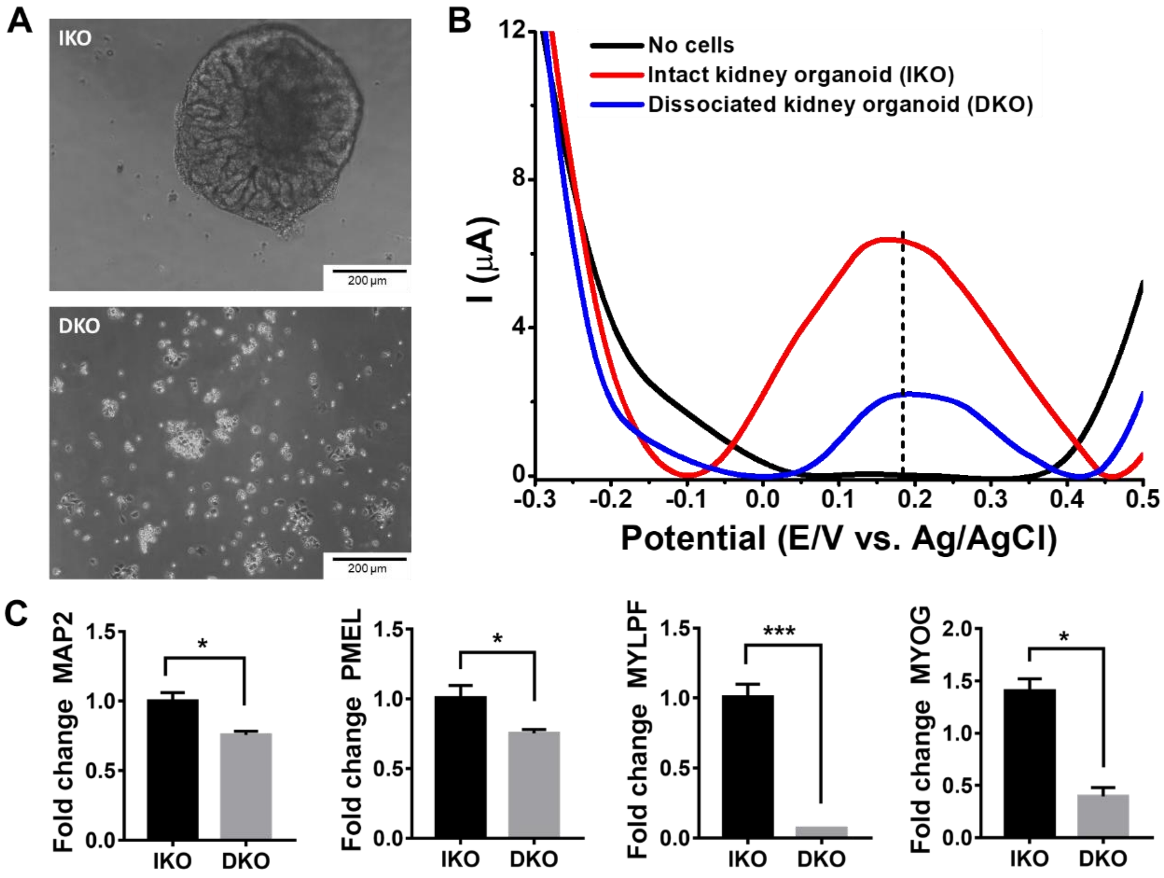

Figure S12. Analysis of kidney organoids and dissociated organoids. (A) Optical microscopic images and (B) DPV signals of intact kidney organoids (IKO) and dissociated kidney organoids (DKO), respectively. (C) qPCR data of genes related to kidney organoid off-target cells (MAP2: neuronal cell marker, PMEL: melanoma cell marker, MYLPF & MYOG: muscle cell marker).

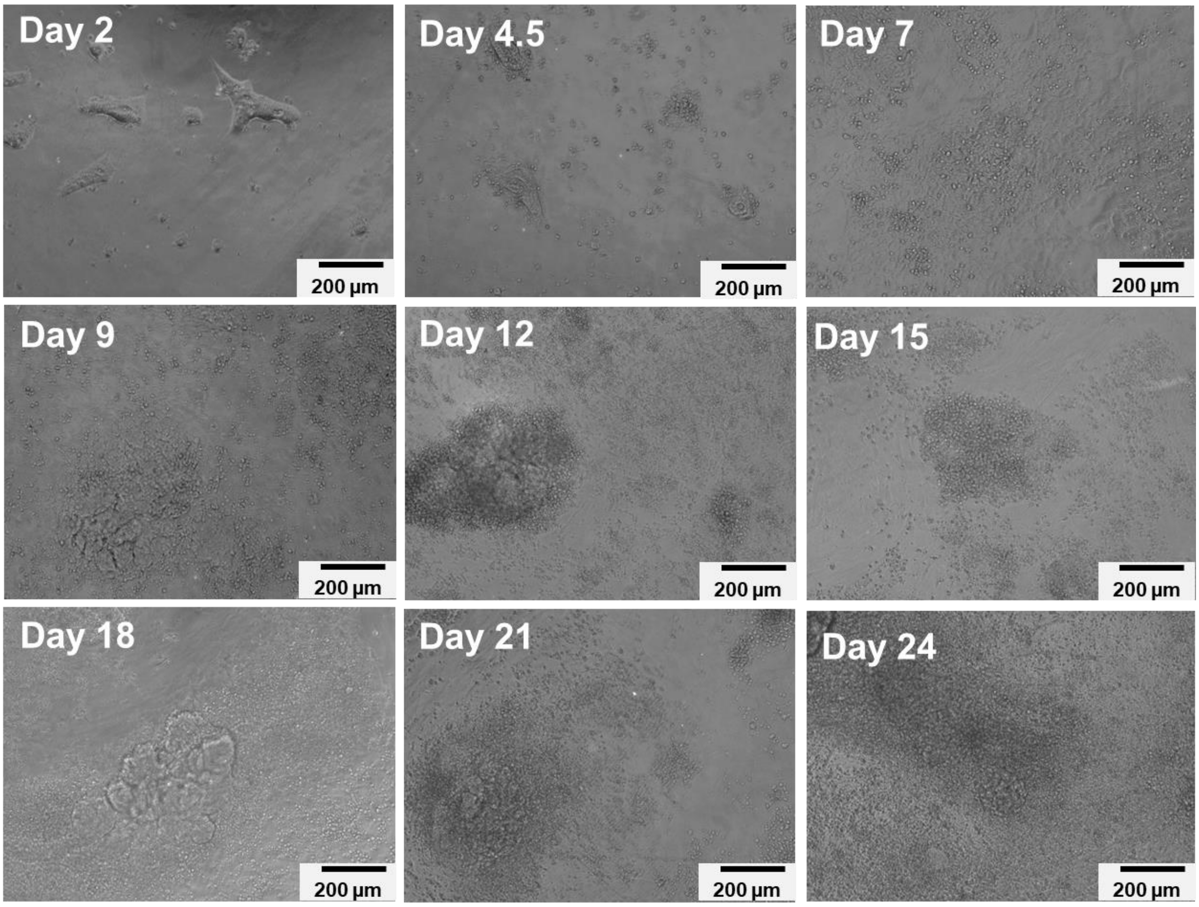

Figure S13. Time-dependent optical images of hiPSC differentiation into kidney organoids.

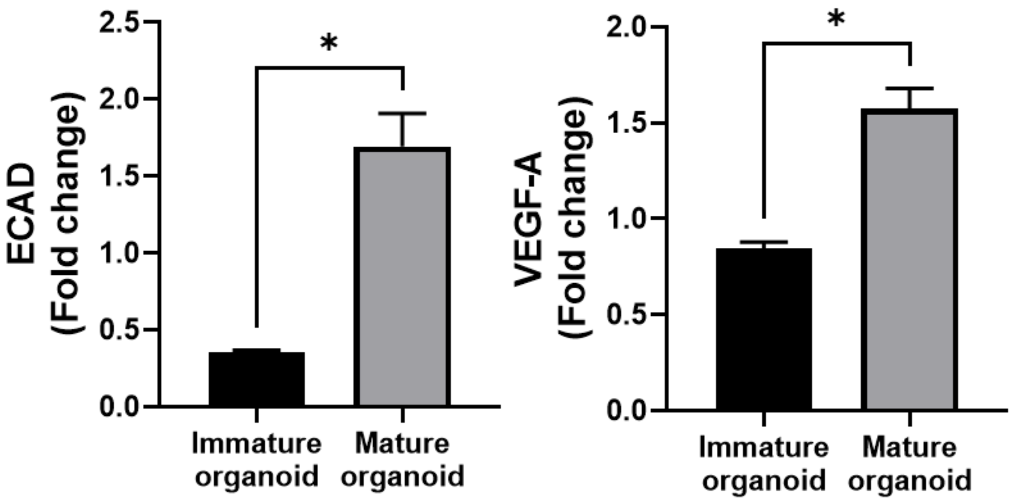

Figure S14. Expression of distal tubule (ECAD) and vascular endothelial cell (VEGF-A) markers in kidney organoid samples.

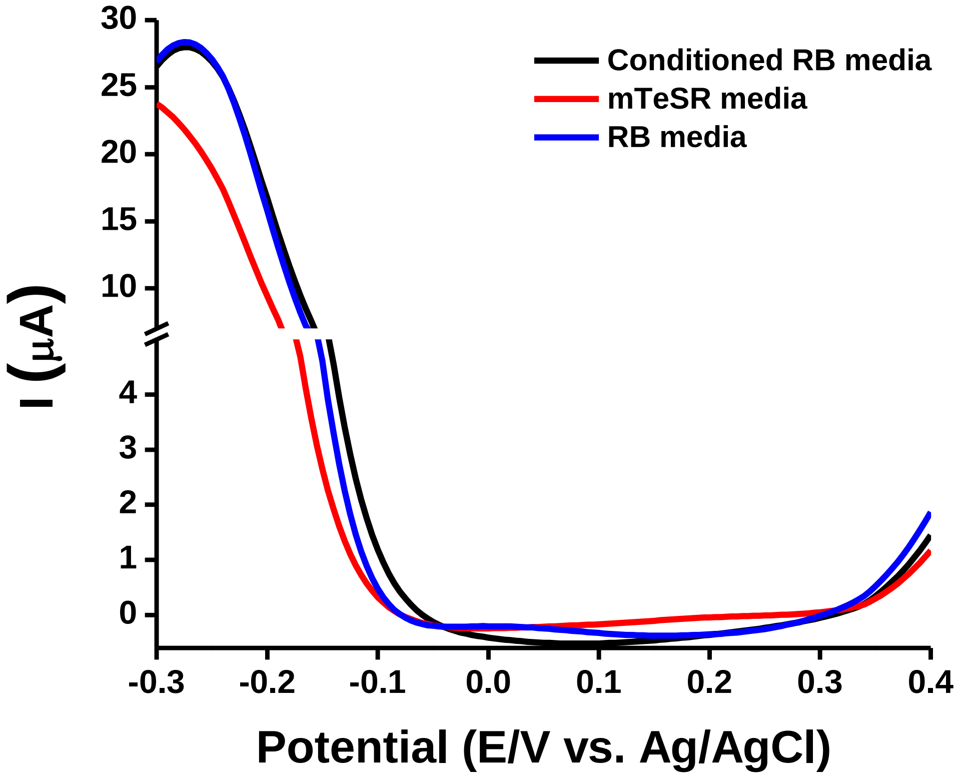

Figure S15. DPV detection of culture media used in all experiments (mTeSR media, RB media, and conditioned RB media).

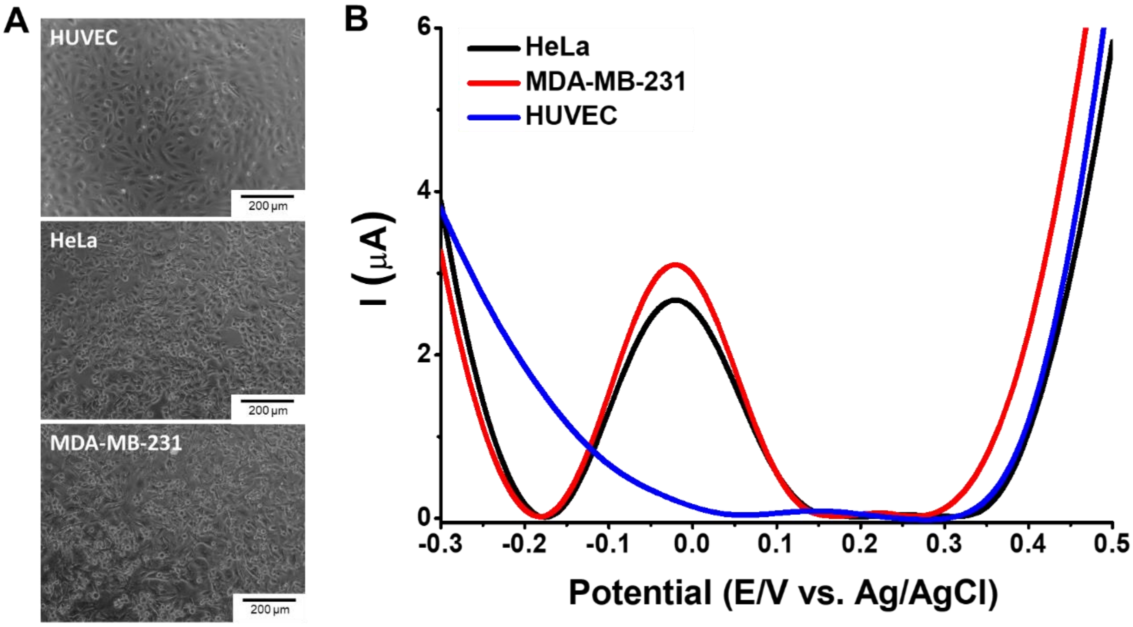

Figure S16. (A) Optical images and (B) DPV detection of human cancer cells (HeLa and MDA-MB-231) and normal vascular cells (HUVEC).

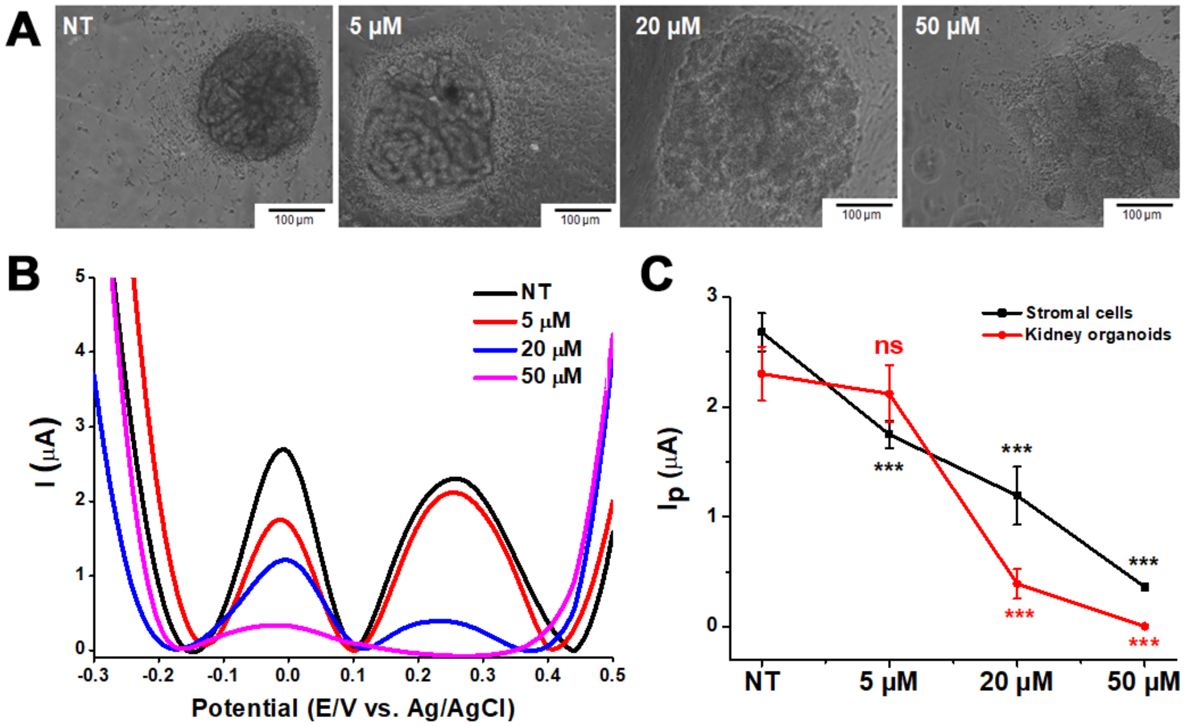

Figure S17. Initial study of kidney organoid on the HCGN platform for drug screening applications. (A) Optical microscopic images, (B) DPV voltammogram, and (C) calculated I_p value from kidney organoid samples under no drug (NT) and nedaplatin treatment at concentrations of 5, 20, and 50 μM (Student's *t*-test, n = 3, ***p<0.001, ns = not significant).

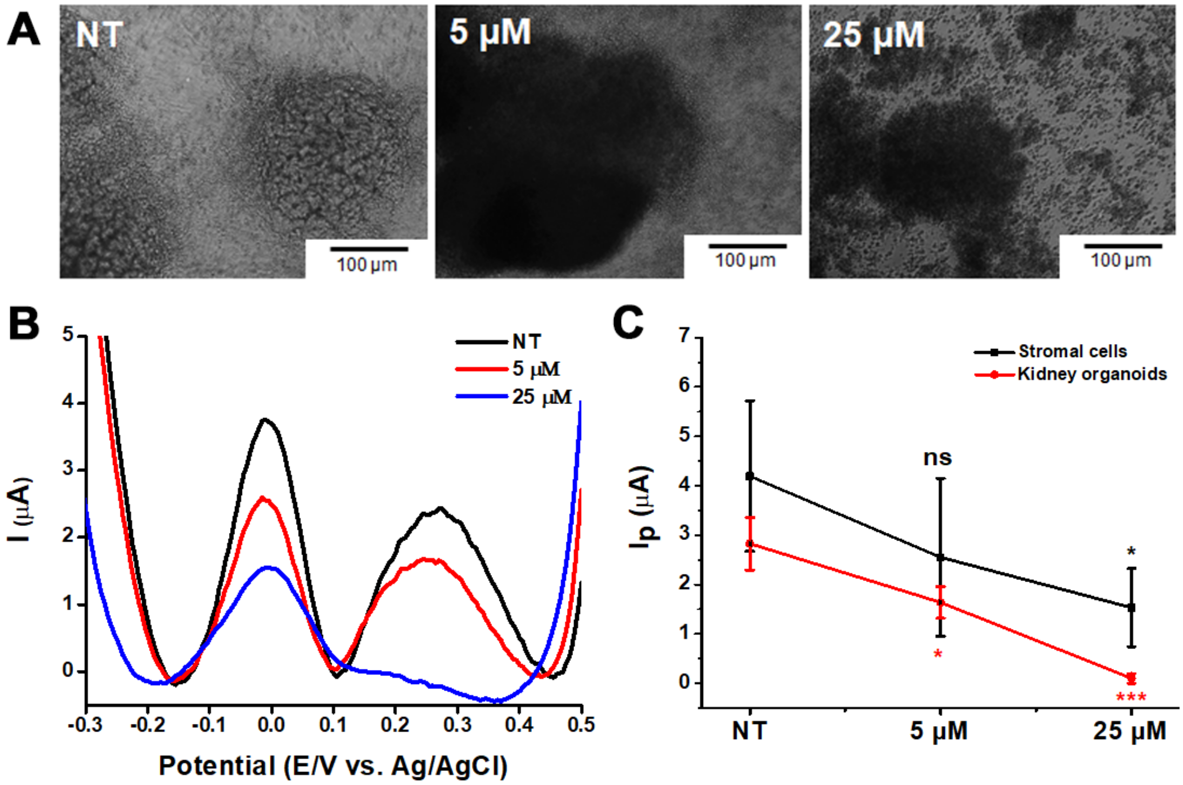

Figure S18. Initial study of kidney organoid on the HCGN platform for drug screening applications. (A) Optical microscopic images, (B) DPV voltammogram, and (C) calculated I_p value from kidney organoid samples under no drug (NT) and cisplatin treatment at concentrations of 5 and 25 µM (Student's *t*-test, n = 3, *p<0.05, ***p<0.001, ns = not significant).
